# High activity levels of avian influenza upwards 2018–2022: A global epidemiological overview of fowl and human infections

**DOI:** 10.1016/j.onehlt.2023.100511

**Published:** 2023-02-20

**Authors:** Yanxia Sun, Ting Zhang, Xiang Zhao, Jie Qian, Mingyue Jiang, Mengmeng Jia, Yunshao Xu, Weizhong Yang, Luzhao Feng

**Affiliations:** a School of Population Medicine and Public Health, Chinese Academy of Medical Science & Peking Union Medical College, Beijing, China; b Department of Influenza, WHO Collaborating Centre for Reference and Research on Influenza, Chinese National Influenza Centre, National Institute for Viral Disease Control and Prevention, Chinese Centre for Disease Control and Prevention, Beijing, China

**Keywords:** Avian influenza, Global epidemiological overview, Poultry infection, Human infection, One health

## Abstract

Due to growing activities of avian influenza, more attention should be paid to avian influenza virus infections. Global summaries or national reports lack data on epidemiological patterns of avian influenza. A descriptive epidemiological analysis of avian influenza outbreaks from 2018 to 2022 was conducted, particularly fowl infections, human infections, and sequence alterations. The number of fowl infection outbreaks in the first half of 2022 was the highest level in the five-year period. Countries or regions could reliably be classified into three clusters according to fowl infection activity scores, with 60.0% of countries or regions in C1 in Europe. Additionally, two host infection patterns of countries were noted, led by the Taiwan (China) region and Germany. Human infections also increased, with 88.1% of cases being in China with an increasing risk of cases in northern China. Sequences that were furin cleavage motif present spread from Asia to Europe and North America over the five-year period. Continuous changes in the global activities of avian influenza highlight the need for sustained global surveillance, including strengthening monitoring capacity for vulnerable population and dynamically detecting new cases or genetic variations of the avian influenza virus under the One Health framework.

## Highlights

• A descriptive epidemiological landscape of avian influenza outbreaks from 2018 to 2022.
• Increasing activity level of avian influenza during and after the COVID-19 pandemic.
• Countries classified into three clusters by fowl infection activity scores.
• Sequences of avian influenza viruses with furin cleavage motif present spread.
• Consistent surveillance and genetic data-sharing needed urgently.

## 1 Introduction

Public health interventions are critical in fighting influenza A viruses as novel outbreaks are possible. Depending on the host of origin, influenza A viruses can be classified as avian, swine, or other animal influenza viruses. Phylogenetically, all mammalian influenza viruses are derived from avian influenza viruses (AIVs), and the earliest recorded history of these viruses dates back to 1878 [1,2]. Some subtypes of AIVs, such as highly pathogenic avian influenzas (HPAI) with hemagglutinin (HA) glycoproteins H5 and H7, are capable of causing major mortality events in birds. However, the majority of AIV subtypes cause asymptomatic or mild infections, complicating animal virus control [3]. Outbreaks of AIVs pose serious threats to animal safety and human security as well as cause significant economic losses worldwide [4]. For instance, in late February 2003, the Netherlands experienced an outbreak of the H7N7 AIV, which affected 255 farms, culling >30 million poultry. Since 2004, H5N1 AIV infections have been reported in East Asia, Southeast Asia, and Europe. Moreover, five waves of H7N9 infections occurred in mainland China from 2013 to 2017. As of September 27, 2017, the total number of officially reported laboratory-confirmed cases and human deaths were 1533 and 607, respectively [5]. Recurrent human infections with AIVs increase the chance of viral recombination, mutation, or both, leading to human-to-human transmission [6].

With an increased focus on animal and human health issues, the United Nations System Coordinator for Combating Avian and Human Influenza (UNSIC) has worked with the World Health Organization (WHO) and several international organizations to develop a strategic framework for reducing the risk of emerging zoonotic diseases. National agencies are working together to conduct and promote extensive surveillance, rapid testing, and sequence sharing [7]. Live poultry in both wholesale and retail markets are thought to be incubators of novel influenza virus subtypes, and low pathogenic viruses may be undetectable in these facilities and markets without active surveillance [[8], [9], [10]]. Additionally, poultry vaccinations may mitigate infection risks, and the sharing of genetic sequence data of AIVs among WHO member countries may improve virus control and aid in early responses to novel influenza viruses.

With the COVID-19 pandemic and the implementation of social and individual prevention measures, seasonal influenza activity has declined. However, infections with zoonotic influenza viruses have not appeared to decrease, suggesting that additional attention to avian influenza may still be required. Global summaries and national reports lack data on epidemiological patterns of avian influenza. Therefore, we performed a descriptive epidemiological analysis of fowl infection outbreaks, human infection cases, and genetic changes in major AIVs between 2018 and 2022. Spatiotemporal analysis of public data pooling animal, human, and viral sequences provided data for risk assessments of avian influenza outbreaks and preparations for future pandemics.

## 2 Methods

### 2.1 Data sources

We obtained data on 2018–2022 HPAI fowl infection outbreaks from multiple public websites, including the Ministry of Agriculture and Rural Affairs of the People's Republic of China (http://www.moa.gov.cn), China Animal Health and Epidemiology Center (https://www.cahec.cn), and the Centre for Health Protection (https://www.chp.gov.hk/en/index.html), which collected data from the WHO website and Food and Agriculture Organization Emergency Prevention System for Animal Health (FAO EMPRES-AH). Fowl infections were summarized by time, place, host type (domestic/wild), and AIV subtype. For human infection cases, we obtained individual demographic data from the Center for Health Protection. Data on human infections with avian influenza virus subtypes were summarized by person, place, and time. Additionally, data for 2022 were collected through June 30.

### 2.2 Avian influenza activity level definitions

Given the wide variation in the number of HPAI fowl infection outbreaks by year, we used the proportion of general fowl infection outbreaks for further analysis. Based on proportions from 2018 to 2022, we divided all the countries or regions into C1 (<0.01), C2 (0.01–0.1) and C3 (>0.1) clusters. Spearman's correlations between avian influenza activity of each of the two proportions were calculated to indicate the interactions of fowl infections between regions. Significant correlations (*P* < 0.05) were visualized using a heatmap.

### 2.3 AIV sequences collection

We collected information on the H5N1, H5N6, and H5N8 sequences from the GISAID Initiative database. The NextStrain workflow was used to calculate the divergence from the root as well as genetic changes of each sequence [11]. Wilcoxon tests were used to explore divergence changes between sequences in adjacent two-year pairs.

### 2.4 Statistical analysis

Descriptive statistics and statistical analyses were conducted using RStudio (Version 1.4.1717). Prism 9 (Version 9.1.1) and RStudio were used to plot figures.

## 3 Results

### 3.1 Overview of fowl infections with HPAI viruses

**Fig. 1:**
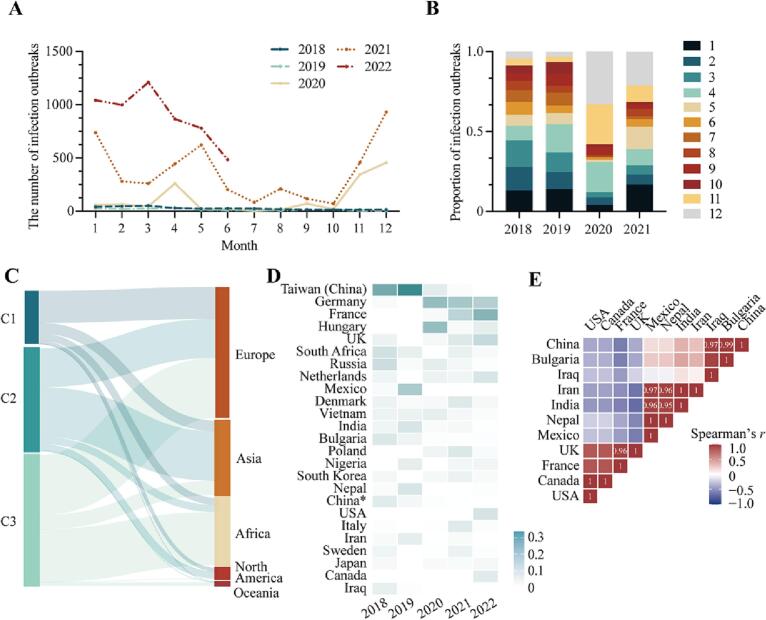
Temporal and spatial analysis of fowl infections with HPAI viruses around world from 2018 to 2022. (A) The number of fowl infection outbreaks displayed by month. (B) The proportion of fowl infection outbreaks reported in each month. (C) Sankey diagram showing distribution of countries or regions from different avian influenza activity level (C1–C3). (D) Heatmap showing proportions of fowl infection outbreaks each year. Countries or regions with top 25 total proportions are shown. Due to different patterns of outbreaks from other regions in China, the Taiwan region is listed separately. (E) Heatmap showing significant Spearman correlation coefficients between proportions of fowl infection outbreaks in different countries. *P* < 0.05 is used as the cutoff value.

We collected data on global fowl infection outbreaks caused by HPAI viruses from 2018 to 2022. In total, a minimum of 312, 187, 1377, and 4407 fowl infection outbreaks were reported worldwide respectively. Notably, the number of outbreaks from January to June 2022 surpassed that of the entire year of 2021, with 5380 outbreaks and nearly 17 million bird infections reported. The number of outbreaks in each month of both 2021 and 2022 was higher than that in the same month of the previous years (Fig. 1A). Additionally, seasonal occurrences of outbreaks have varied. In 2018 and 2019, outbreaks from January to April accounted for the majority of infections, while a sharp increase was noted in November and December of 2020. In 2021, on the other hand, the proportion of infections was significantly higher in May compared with previous years, in addition to the spring and winter months (Fig. 1B).

Regarding the distribution of outbreaks worldwide, 38, 26, 36, 64, and at least 59 countries or regions reported outbreaks from 2018 to 2022, with the majority occurring in Europe, North America, and Asia (Supplementary Fig. 1). We divided the overall activities of countries and regions into three clusters (C1–C3) based on the proportion of avian infection outbreaks over the five-year period We found that 60.0% (9/15) of C1 countries were in Europe, while 48.3% (14/29) and 37.9% (11/29) of C2 regions were in Asia and Europe, respectively (Fig. 1C). The top 25 countries or regions with fowl infections are shown in Fig. 1D. Of these countries, the Taiwan (China) region had the most outbreaks during the years 2018–2019, while outbreaks in Germany, France, and Hungary started increasing in 2020. We also explored the co-occurrence of outbreaks in countries and regions (Fig. 1E) and found several instances, such as with the US and Canada; the United Kingdom and France; China, Iraq, and Bulgaria; and Iran, Nepal, India, and Mexico. These partner co-occurrences may be explained by the relative geographic proximity of these countries or bird migration routes [12].

**Fig. 2:**
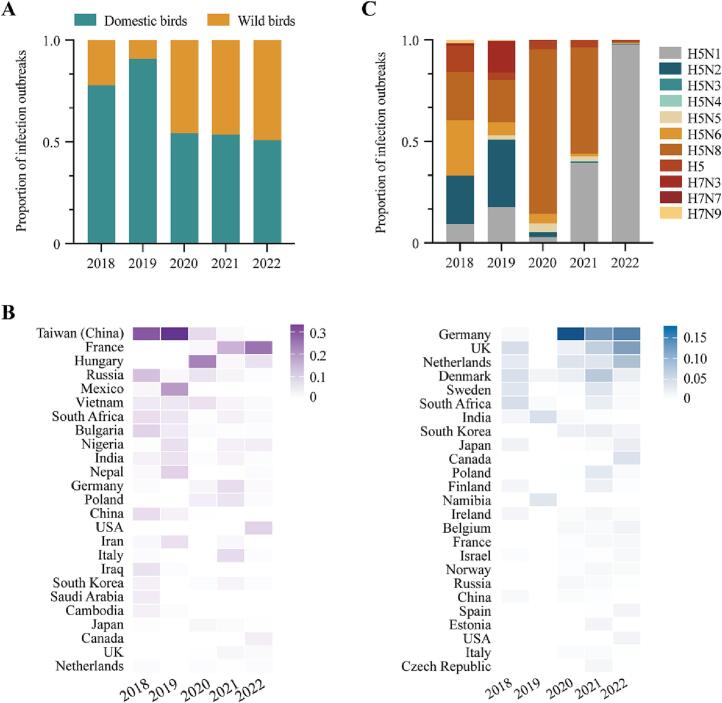
Temporal and spatial analysis of HPAI infections of domestic bird s and wild birds from 2018 to 2022. (A) The proportion of fowl infection outbreaks by host type. (B) Heatmap showing proportions of domestic bird outbreaks (left panel) and wild bird outbreaks (right panel). Top 25 countries or regions are shown. (C) The proportion of fowl infections by virus subtypes.

Avian influenza hosts are primarily divided into two types: domestic and wild birds. In 2018 and 2019, the proportion of domestic bird infections was considerably higher than that of wild bird infections. However, the ratio began to equalize in 2020 (Fig. 2A). We also examined the top 25 countries or regions for domestic bird infections and wild bird infections (Fig. 2B) and found that outbreaks in the Taiwan (China) region, France, and Hungary were dominated by domestic bird hosts, whereas outbreaks in Germany, the United Kingdom, and the Netherlands were dominated by wild bird hosts.

The dominant subtypes of viruses infecting fowl changed rapidly from 2018 to 2022. In 2018, H5N2, H5N6, and H5N8 were the most common subtypes. In 2019, the H5N2 subtype was dominant. From 2020 to the first half of 2021, H5N8 subtype became the dominant subtype. Finally, from the second half of 2021 to the present, the H5N1 subtype was most common (Fig. 2C). These findings indicate that consistent and close surveillance is needed to detect varying dominant AIV subtypes earlier.

### 3.2 Overview of human infections with avian influenza viruses

**Fig. 3:**
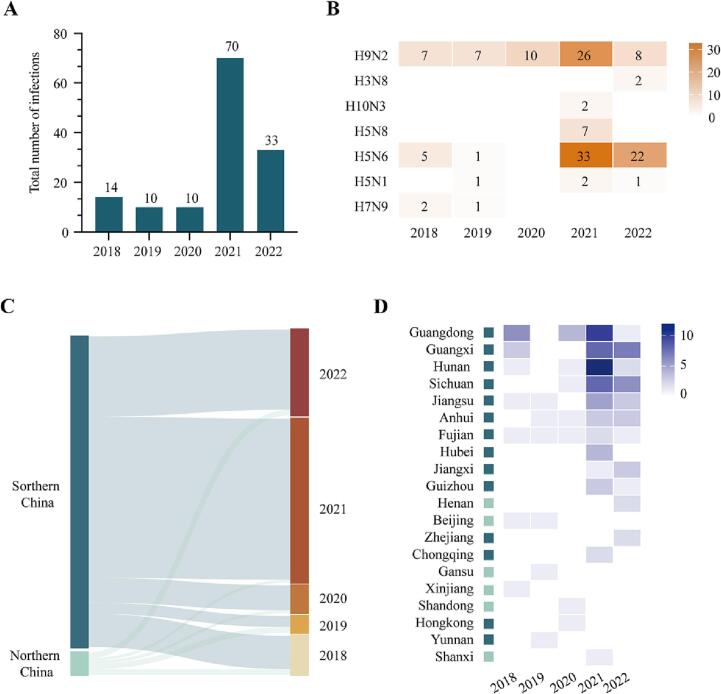
Temporal and spatial analysis of human infections around world from 2018 to 2022. (A) The number of human infection cases by year. (B) Cases of human infections by virus subtypes. (C) Sankey diagram showing distribution of human infections in China. (D) Heatmap showing the number of human infection cases by province in China each year. Provinces with dark or light green box are in southern or northern China, respectively. (For interpretation of the references to colour in this figure legend, the reader is referred to the web version of this article.)

As a zoonotic infectious disease, AIVs can also infect vulnerable individuals who are exposed to virus-carrying fowl. We obtained infection data from 137 individuals and demographic information from public sources, including 14, 10, 10, 70, and 33 cases so far in 2018, 2019, 2020, 2021, and 2022, respectively (Table.1; Fig. 3A). Of these individuals, 88.1% (126/137) were from China. Children aged <10 years accounted for 39.2% (56/137) of the human infections, suggesting that children with underdeveloped immune systems may require further attention. Additionally, the H9N2 and H5N6 subtypes accounted for 44.8% (64/137) and 42.7% (61/137) of the infections, respectively, with the dominant subtype varying by year (Fig. 3B). From 2018 to 2020, H9N2 prevailed considerably. However, the number of patients infected by the H5N6 virus subsequently and rapidly increased, exceeding H9N2 cases in 2021 and 2022.

**Table 1:** Demographic information of AIV human infection cases.

		Number	%
Country			
	China	126	88.1%
	Russia	7	4.9%
	India	2	1.4%
	Laos	2	1.4%
	Cambodia	2	1.4%
	the US.	1	0.7%
	Nepal	1	0.7%
	Senegal	1	0.7%
	Oman	1	0.7%
Virus type			
	H9N2	64	44.8%
	H5N6	61	42.7%
	H5N8	7	4.9%
	H5N1	4	2.8%
	H7N9	3	2.1%
	H3N8	2	1.4%
	H10N3	2	1.4%
Gender			
	Male	66	46.2%
	Female	61	42.7%
	Unknown	16	11.2%
Age			
	≤10	56	39.2%
	11–40	15	10.5%
	41–60	40	28.0%
	>60	14	9.8%
	Unknown	18	12.6%
Survival			
	Yes	110	76.9%
	No	18	12.6%
	Unknown	15	10.5%

As the vast majority of human infection cases were in China, we further analyzed the country's spatial and temporal distribution of cases (Fig. 3C). Overall, H9N2 and H5N6 subtypes both accounted for 47.6% (60/126) of the infections of China and 93.3% of the cases were in southern China. However, an increasing risk of cases in northern China was noted, particularly in Henan province. This province had not reported a human case since 2018; however, two cases were then reported in 2022, including infections with the H3N8 subtype. Guangdong, Guangxi, Hunan, and Sichuan provinces in southern China had high numbers of cases the past two years, and Jiangxi, Zhejiang, and Henan provinces may require further focus (Fig. 3D).

### 3.3 Genetic changes of H5N1, H5N6, and H5N8 subtypes

**Fig. 4:**
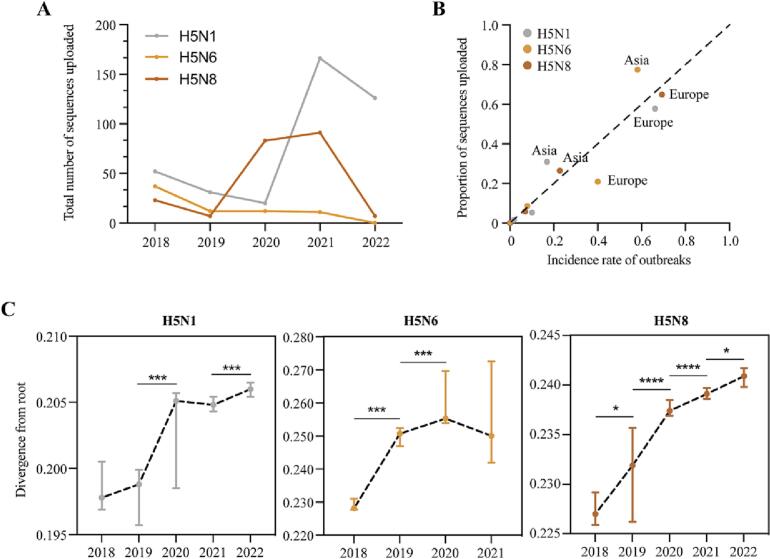
Sequence changes of H5N1, H5N6 and H5N8 subtypes during 2018 to 2022. (A) The number of avian virus sequences uploaded to GISAID database. (B) Comparison of the proportion of sequences uploaded in different continents with that of infection cases. (C) The divergence of sequences from root each year. *P* values were derived from two-sided Wilcoxon test; *P < 0.05, **P < 0.01, ***P < 0.001, ****P < 0.0001.

The H5N1, H5N6, and H5N8 subtypes were the primary highly pathogenic virus subtypes causing infections. We investigated the genetic changes in the H5N1, H5N6, and H5N8 sequences uploaded to the GISAID database. The number of virus sequences of different subtypes showed a trend consistent with the number of infections (Fig. 4A). We then compared the proportion of sequences uploaded in different regions with that of the infection cases (Fig. 4B). Compared with Asian countries, European countries were underrepresented in sequence sharing, suggesting that the promotion of public sequence sharing may need to be furthered.

From 2018 to 2022, all H5N6 and H5N8 sequences uploaded were assigned to clade 2.3.4.4. For the H5N1 subtype, the number of sequences in clade 2.3.4.4 increased starting in 2021, completely suppressing clades 2.3.2.1a and 2.3.2.1c (Supplementary Fig. 2). We subsequently examined the divergence of each sequence from its root (Fig. 4C). For H5N1 and H5N8, the divergence of sequences increased steadily each year, and sequences in 2022 were more divergent than those in 2021. However, H5N6 sequences showed relatively low divergence in 2021, which was consistent with the prevalence of AIV subtypes. Furin plays an important role in the viral life cycle during highly pathogenic influenza A virus infections. Furin-dependent proteolytic activation of highly pathogenic H5 and H7 haemagglutinin precursor subtypes is critical for the production of fusion-competent infectious virions [13,14]. All 72 sequences of the H5N6 subtype were furin cleavage motif-present, and only one sequence (1/211) of the H5N8 subtype was motif-absent. The proportion of H5N1 subtype sequences lacking the furin cleavage motif was approximately 11.6%. No significant differences in the proportions across years were noted; however, regions of sequences reported spread from Asian to European and North American countries.

## 4 Discussion

We conducted descriptive epidemiological analyses of avian influenza outbreaks from 2018 to 2022, particularly regarding fowl infections, human infections, and sequence alterations. The epidemiology of variant viruses varied across time series, geographical regions, and subtypes. Several key points were observed. Based on the historical timeline, the COVID-19 pandemic has been associated with a notable decrease in seasonal influenza infections worldwide compared to previous flu seasons. In contrast, the risk of avian influenza infections has increased during the COVID-19 pandemic. Between 2018 and the first half of 2022, the number of avian influenza fowl infections significantly increased, with the first half of 2022 exceeding every previous year in numbers. Regarding human AIV infections, which were primarily reported in China, during 2021 and the first half of 2022, the human infections increased over 2018, 2019, and 2020. The trend was also evident in Europe, with the European Food Safety Authority and European CDC declaring the 2021–2022 HPAI epizootic as the largest epidemic to have occurred in Europe so far. These findings suggest that the prevention and control of avian influenza is vital [15].

In analyzing spatial distributions, we found that the key areas showing domestic bird, wild bird, and human infections with HPAI varied. Globally, domestic bird outbreaks were primarily found in Taiwan (China), France, Hungary, Russia, Mexico, and Vietnam, whereas those with wild bird were primarily found in Germany, the United Kingdom, the Netherlands, Denmark, Sweden, and South Arica, showing two inconsistent patterns of distribution. Wild birds may have carried viruses, such as AIV, from the original outbreak site to other places with poultry and humans. In a US study conducted by Bevins, H5N1 HPAI viruses were isolated from wild waterfowl from two Atlantic coastal states, coinciding with the movement of banded birds within the Atlantic flyway [12,16]. Most human infections with AIV (88.1%) were reported in China. Previous studies reported that the internal genes of H9N2 subtype can be exchanged with other AIVs, which may increase influenza pandemic risk [17,18]. Southern China was more likely to experience a bird flu epidemic than northern China, which may have corresponded to the prevalence of bird flu, temperatures, and humidity levels [19]. Relatively stable temperatures and high humidity levels are suitable for the spread and survival of bird flu. However, the risk areas of human infection with avian influenza may also be expanding.

Additionally, the proportion of infected domestic birds gradually decreased, while human infections showed a steadily rising trend. Positive active virological surveillance can detect variations across hosts. One study in China found that HPAI H5N1 caused a higher positive rate of infections in ducks in southern China, whereas in northern China, the majority of hosts were chickens. The virulence of influenza viruses is determined by multiple factors, including both viral and host factors. Domestic and wild bird distributions vary due to national highways, distances to nearby lakes and wetlands, and human population densities. These factors are related to AIV infection outbreaks as well as to prevention and control strategies. In addition, vaccination plays a crucial role in preventing H5N8 infections in poultry in China. From September 2020 to June 2021 in China, the H5N8 virus was detected in wild birds, but no disease outbreaks in domestic birds were observed. Simultaneously, in Japan and South Korea, more than two million domestic birds were killed by the H5N8 virus [20]. The global spread and reassortment of different local low pathogenic viruses may have increased the viral multiplicity of clade 2.3.4.4 viruses, occurring substantially faster and higher rates than those observed in other H5 clade virus types [21]. Clade 2.3.4.4 viruses were dominant among H5N1, H5N6, and H5N8 subtypes, and regions containing sequences with a furin cleavage motif present spread from Asia to Europe and North America over the five-year period, suggesting increased activities of highly pathogenic AIV.

Worldwide, approximately 75% of zoonotic diseases and 60% of emerging infectious diseases are of animal origins [22]. A range of emerging and re-emerging zoonotic infections significantly impact the lives of humans and animals, placing substantial economic burdens on social development [23,24]. The One Health approach is widely used for the prevention and control of zoonotic diseases; however, challenges in controlling avian influenza remain. The spread and outbreaks of AIV have increased due to global trade, poultry production, climate change, bird migration, human movement, and an increasing global population. Currently, biosecurity and culling are the main recognized control strategies for avian influenza prevention, while vaccination is an optional measure for some countries. Thus, to determine whether poultry vaccination should be implemented, we grouped strategies for avian influenza prevention and control in the One Health framework into two patterns: those with and without vaccination measures in place. Some countries in European or American regions that do not implement poultry vaccination measures and rely on active surveillance and monitoring of poultry and wild birds for avian influenza prevention and control may detect large numbers of outbreaks of poultry infections, indicating that the most vulnerable group for the disease in these places is the poultry population. When an outbreak occurs, culling infected domestic birds provides the primary form of control. In contrast, in China where poultry vaccination measures are in place, the number of poultry outbreaks is significantly reduced by vaccination against H5 and H7 strains, along with active surveillance. In these countries, the human population is the most vulnerable group for the disease. Therefore, different prevention and control strategies provide stronger protection of vulnerable groups, which remains the primary goal for avian influenza prevention and control.

Furthermore, ecosystem health is an important component of the One Health approach and a key factor influencing avian influenza disease infections and outbreaks. Disrupting the balance between humans and nature, such as disrupting or encroaching on the migratory routes of wild birds, may accelerate viral recombination, leading to the emergence of novel avian influenza viruses. From a health perspective, human and animal infections are closely linked to the ecosystem and social drivers. One Health guides us to recognize the interconnectedness of human, animal, and ecosystem health. This may include coordinated, collaborative, multidisciplinary, and cross-sectoral approaches to address potential risks at the animal-human-environment interface. Several leading suggestions for the prevention and control of avian influenza are included in the One Health approach. First, comprehensive surveillance of AIV infections in animals and humans is essential in assessing the risk and guiding the use of control measures [7]. Studies have shown that many avian viral infections can cause mild disease [25]. Information and influencing factors of avian influenza outbreaks in humans and birds can be collected via several effective and sensitive surveillance systems. These tools may help to analyze and reveal the occurrence and development of outbreaks to detect infections in a timely manner. An efficient surveillance system can report a warning earlier to stakeholders to respond in a timely manner. Second, the advancement of public virus sequence sharing is vital. As AIVs may be the progenitors of the next human pandemic virus, full support from the international and scientific community is essential. To fully understand the spread, evolution, characteristics, and pathogenesis of AIVs, broad information sharing about the viruses, including their genetic sequences, should be internationally fostered [26,27].

Pandemics may arise when novel influenza A viruses, derived in whole or in part from animal or avian influenza viruses, adapt to transmit efficiently in a human population with little population immunity to contain onward transmission [28]. Cross-reactive immunity in humans is considered when assessing the threat of a pandemic from a newly emerged animal influenza virus. When there is little antigenic cross-reactivity between novel avian influenza A and the current strain, combined with low seroprevalence of the current strain and high R_0_ of novel avian influenza A, this virus may have significant zoonotic and pandemic potential [29]. Even as other current pandemics are ongoing, avian influenza consideration is required.

Our study had several limitations. First, the detection of infections relied on surveillance and testing. The lack of awareness or testing in some undeveloped regions, as well as low levels of circulation of seasonal influenza due to the ongoing COVID-19 pandemic, might have hindered the early identification of avian influenza infections, leading to underestimation. Therefore, attention should be paid to avian influenza infection and the surveillance capabilities in undeveloped countries or regions should be enhanced to provide support for prevention, control, and risk assessment of avian influenza infection. Second, secondary data were collected from public websites, and therefore could not be assessed for bias. Third, the analysis of genetic sequence alterations did not include specific mutation sites and functional regions, which may need further analysis.

## 5 Conclusions

In conclusion, high activity levels of avian influenza warrant further consideration. In the face of a mixed epidemic of avian influenza and other infectious diseases, an unmet demand worldwide exists for stronger and more dynamic monitoring systems for new cases or genetic variations. Considering the characteristics and interactions of hosts, species, and environments, integrating One Health approaches in prevention, control, and risk assessment systems may be needed.

## Funding

This study was funded by the CAMS Innovation Fund for Medical Sciences (2021-I2M-1-044), Guilin talent mini-highland scientific research project (Municipal Committee Talent Office of Guilin City [2020] No. 3–05) and the Non-profit Central Research Institute Fund of the 10.13039/501100005150Chinese Academy of Medical Sciences (NO: 2021-RC330-002).

## Compliance with ethics guidelines

All authors declare no competing interests.

## Author contributions

**L. Feng:** Conceptualization, Supervision, Funding acquisition. **W. Yang:** Project administration, Supervision, Funding acquisition. **Y. Sun:** Conceptualization, Methodology, Investigation, Writing - Original Draft. **T. Zhang***:* Software Programming, Writing - Original Draft.**X. Zhao:** Formal analysis, Validation. **J. Qian:** Investigation, Data Curation. **M. Jiang:** Validation. **M. Jia:** Writing - Review and Editing. **Y. Xu:** Data Curation.

## Data Availability

Data will be made available on request.

## Appendix A Supplementary data

Supplementary material Image 1
