# Supplementary material for: High activity levels of avian influenza upwards 2018–2022: A global epidemiological overview of fowl and human infections
Source: One Health. 2023 Feb 20;16:100511. doi: 10.1016/j.onehlt.2023.100511 (PMC10288038; doi:10.1016/j.onehlt.2023.100511)
Supplement: Supplementary file 1 — Supplementary material [file mmc1.docx]

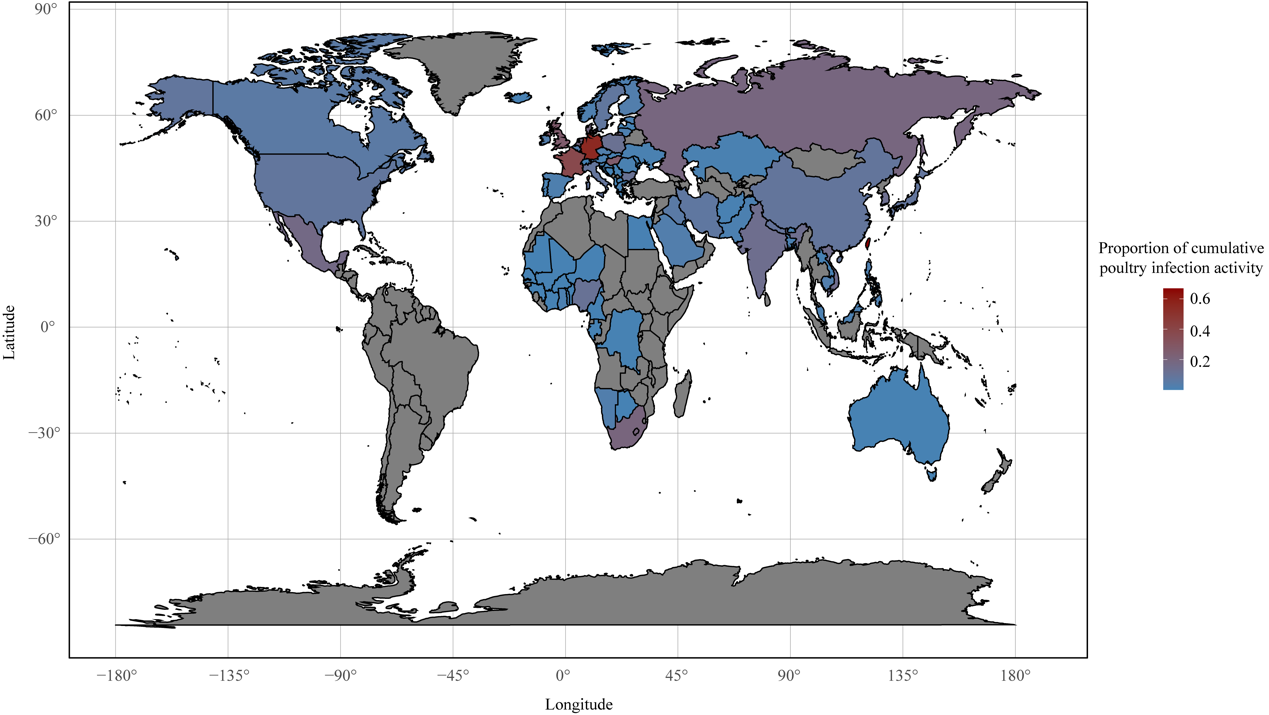


**Supplementary Fig.1. HPAI prevalence of poultry infections around the world from 2018-2022.**


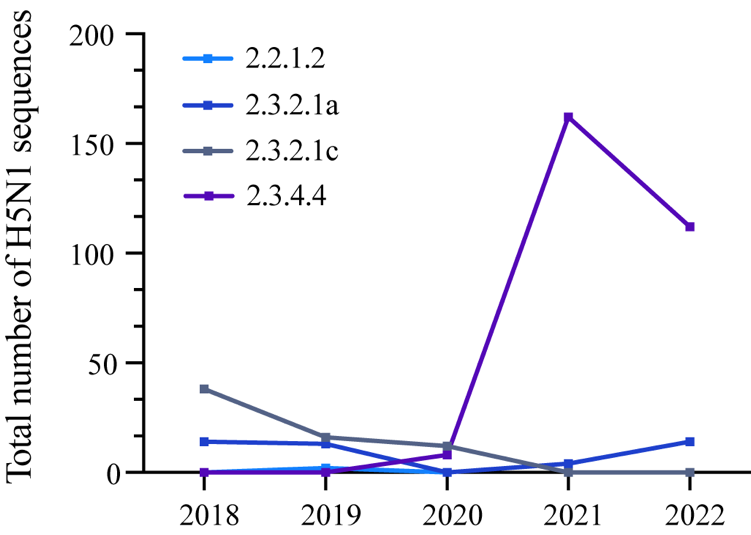


**Supplementary Fig.2. The number of H5N1 virus sequences uploaded to GISAID database by virus clade.**
